# Clinico-Immunological Analysis of Eggplant (*Solanum melongena*) Allergy Indicates Preponderance of Allergens in the Peel

**DOI:** 10.1097/WOX.0b013e3181b71c07

**Published:** 2009-09-15

**Authors:** Bheemanapalli N Harish Babu, Yeldur P Venkatesh

**Affiliations:** 1 Department of Biochemistry and Nutrition, Central Food Technological Research Institute (CSIR Laboratory), Mysore, Karnataka State, India

**Keywords:** allergen, eggplant, food allergy, peel, simulated gastric fluid

## Abstract

**Background:**

Eggplant (*Solanum melongena *L.) is known to cause food allergy in some Asian countries but detailed studies on eggplant allergy are lacking.

**Objective:**

The objective is to investigate sensitization to different parts of eggplant fruit, and detection of the allergens.

**Methods:**

Six eggplant-allergic subjects were assessed for sensitization to eggplant (peel/pulp, and raw/cooked) by skin prick test, allergen-specific IgE, and immunoblots. Allergens were analyzed for glycoprotein nature by staining/lectinoblots, and in vitro stability in simulated gastric fluid.

**Results:**

All the eggplant-sensitized subjects showed positive skin prick test with peel, pulp, raw, and cooked eggplant extracts; allergen-specific IgE to all these was positive. Raw eggplant contains 5 allergens in the range 36-71 kD. Most allergens are localized in the eggplant peel (9 allergens; 26-71 kD range) than the pulp (3 allergens; 52-71 kD); among these, the 26, 28, 36, and 71 kD allergens seem to be heat-stable. The 43, 45, 64, and 71 kD allergens are detected as glycoproteins; the 26, 64, and 71 kD allergens are stable displaying retention of IgE-binding ability in simulated gastric fluid digestion.

**Conclusions:**

Eggplant is a multiallergenic vegetable in the context of presence of allergens in all edible parts of eggplant having preponderance in the peel.

## 

Sensitization to foods varies in different countries reflecting a possible interaction of genetic factors, cultural and dietary habits, and/or exposure to new allergenic sources early in life. The vegetables of the nightshade family (So-lanaceae)--tomato, potato, bell pepper, and eggplant are commonly used in various culinary preparations; in addition, tomato and bell pepper are also consumed in raw form. Eggplant or aubergine (*Solanum melongena *L.) has been cultivated in Asia, Europe, and other parts of the world [1,2]; it is often referred to as the 'king of vegetables' and 'poor man's meat.' In Indian and South African English, eggplant is known as brinjal. Eggplant is also used in traditional medicines [3].

Allergic reactions to Solanaceous vegetables (potato, tomato, and bell pepper) have been well documented and several Immunoglobulin E (IgE)-binding components were identified. Raw potato has been described as a 'multiallergenic' vegetable based on the identification of wide array of allergenic proteins (Sola t 1: patatin, Sola t 2 to Sola t 4: protease inhibitors) [4-6]. In the case of tomato, Lyc e 1 (profilin), Lyc e 2 (*β*-fructofuranosidase), and Lyc e 3 (lipid transfer protein) have been described as major allergens, whereas polygalacturonase 2A and pectinesterase seem to be minor allergens [7-10]. A thaumatin-like protein homologue (Cap a 1) and a 20 kD prohevein-like protein have been identified as major allergens, whereas profilin (Cap a 2), *β*-1,3-glucanase, and L-ascorbate peroxidase have been reported as cross-reactive allergens in bell pepper [10,11].

In recent years, allergic reactions to eggplant have been reported mainly from the Asian region [12-15]. Severe allergic reactions are rather rare [13,16] and the actual prevalence of IgE-mediated eggplant allergy in a study population of 741 subjects is known to be 0.8% with a female predominance [15]. Generally, eggplant is consumed in cooked form. The major symptoms experienced in eggplant allergy include skin rashes, angioedema, and wheezing. In addition, asymptomatic sensitization and nonallergic food hypersensitivity (food intolerance) were also reported to be the causes for some of the adverse reactions to eggplant [15] similar to those seen in the case of tomato in the Mediterranean coast of Spain [17]. To date, only some IgE-binding proteins have been detected from eggplant fruit using sera from a few eggplant-allergic subjects,[12,13] and virtually nothing is known about the number and distribution of allergens in the eggplant fruit. In this study, an attempt has been made to study the difference in sensitization to different parts of eggplant fruit (peel and pulp), and raw versus cooked eggplant fruit to detect the IgE-binding components in the peel and pulp of eggplant fruit. Some characteristics of the eggplant IgE-binding proteins in relation to the presence of glycans and their stability in simulated gastric fluid were assessed by protein analysis and IgE-immunoblotting.

## Materials and method

### Selection of Subjects

This study was undertaken after approval by the Institutional Ethics Committee. Informed consent was obtained from all the study subjects. Six subjects sensitized to eggplant fruit were selected from our recent study on the prevalence of eggplant allergy based on their positive case history, positive skin prick test (SPT), and the presence of serum allergen-specific IgE to eggplant fruit extract [15].

The clinical characteristics of the 6 eggplant-allergic subjects are presented in Table 1. The symptoms of eggplant allergy commonly experienced, because of ingestion of eggplant in cooked form, include skin rashes, itching and swelling in the throat, and itching and reddening of eyes. Two subjects (A3 and A5) had sensitization to eggplant since early childhood. Subjects A1, A2, and A5 have also other food allergies. Four subjects (A1, A2, A3, and A5) have history of other atopic conditions, mostly respiratory allergies like asthma. However, there was no family history of eggplant allergy in any of the eggplant-sensitized subjects.

**Table 1:** Clinical Characteristics of Eggplant-Allergic Subjects

Subject's ID*	Sex/Age (y)	Age at Onset of Eggplant Allergy	Reaction to Eggplant†	Duration for Onset of Symptoms	Other Food Allergy‡	Other Atopy§
A1	F/40	32 years	Itching, reddening and edema of the eye	< 15 minutes	T, P, PN	Wh, A.Rh
A2	F/15	10 years	Skin rashes; Itching and swelling of the throat	< 2 hours	B, C	Wh
A3	M/22	Early childhood	Itching and rashes on hands	< 30 minutes	--	Wh
A4	F/23	14 years	Skin rashes and throat itching	< 1 hour	--	--
A5	F/27	Early childhood	Nasal blockage, Skin rashes and throat itching	< 1 hour	P, T, W	Wh, A.Rh
A6	F/26	23 years	Skin rashes, throat itching and mouth dryness	< 2 hours	--	--

*Family history of eggplant allergy was negative in all cases; Dwelling: rural (A1 and A2); urban (A3-A6).

†Type of exposure: ingestion of eggplant (cooked form) in all subjects.

‡T = tomato; P = potato; PN = peanut; B = banana; C = citrus fruits; W = wheat; §Wh = wheezing; A.Rh = allergic rhinitis.

### Preparation of Allergenic Extracts From Different Parts of Eggplant Fruit

Freshly obtained Mysore green variety (*Solanum melongena *var. serpentinum) of eggplant having a slender long appearance was used in this study because of its frequent consumption in this region. Protein quantitation in the extracts was performed by the dye-binding method using bovine serum albumin (BSA) as standard [18].

#### Fruit extract

It was prepared by blending 50 g eggplant berries in a blender using equal volumes of 10 mM sodium phosphate buffer, pH 7.4 containing 140 mM NaCl (phosphate-buffered saline, PBS), 2 mM phenylmethansesulfonyl fluoride (PMSF, a serine protease inhibitor), and 100 mM L-ascorbic acid (a polyphenol oxidase inhibitor to prevent enzymic browning reaction),[19] centrifuged at 5300*g *for 20 minutes, filtered through Whatman no. 1 filter, and then dialyzed using 3.5 kD cutoff membrane against double distilled water. The dialyzed extract was subjected to acetone precipitation to concentrate eggplant proteins. This was done by adding 4 volumes of chilled acetone (- 20°C) to the dialyzed eggplant extract, kept at -20°C for 8 hours, followed by 2 washes with a small volume of chilled acetone. The excess acetone was decanted and the remaining precipitate was evaporated to dryness and the white curd-like precipitate representing protein concentrate was resuspended in a small volume of sterile deionized water and stored at -20°C. Aliquots of this protein concentrate were used for SPT, ELISA, and immunoblot analysis.

#### Cooked eggplant extract

Fifty grams of eggplant berries were cut into slices, boiled in PBS containing 2 mM PMSF and 100 mM L-ascorbic acid for 15 minutes; cooked eggplant along with the liquid medium was homogenized in a blender, and further processed as described for fruit extract. The cooked eggplant extract was very viscous and sticky, and was not easily amenable to filtration or centrifugation; only a small volume of the extract was recovered for various in vivo and in vitro allergy diagnostic tests.

#### Peel extract

Eggplant berries (250 g) were peeled using a stainless steel vegetable peeler to obtain the green thin skin (peel; 32 g by fresh weight), and was homogenized in a blender using equal volumes of PBS containing 2 mM PMSF and L-ascorbic acid as described for the preparation of fruit extract. Depending on the size of the eggplant fruit and different batches of commercial eggplant (green long slender type), the amount of peel obtained varied from 7 to 13% on a fresh weight basis of eggplant fruit.

#### Pulp extract

The peeled eggplant berries (50 g) from the previous step were used to prepare the pulp extract as described for fruit extract. The pulp also contained some fresh seeds interspersed in the pulp, and it was not practically feasible to separate the tiny seeds from the pulp. Hence, the pulp extract denotes extract obtained from the pulp and the seeds.

### SPT and Prick-By-Prick Test

For SPT, different extracts (prepared without the use of PMSF) were glycerinated to a final concentration of 50% (vol/vol) and the protein content of the extracts was adjusted to 0.5 mg/mL. SPT was performed as per the standard procedure [20] using sterile prick lancetter (Bayer Pharmaceutical Division, Spokane, WA). Histamine.2HCl equivalent to 1 mg/mL histamine base and 50% glycerinated PBS were used as positive and negative controls, respectively. The wheal/flare diameters were measured after 20 minutes. A wheal diameter of > 3 mm compared with the negative control was considered as positive prick test. Prick-by-prick test was carried out as described in the literature [21].

### Detection of Allergen-Specific IgE

Detection of allergen-specific IgE by ELISA [22] was carried out using sera from 6 eggplant-allergic subjects. Briefly, the microtiter wells (Maxisorp; Nunc, Roskilde, Denmark) were coated at 4°C overnight with extracts of eggplant peel, pulp, raw fruit, and cooked fruit (~25 *μ*g protein/well) that had been dialyzed using 3.5 kD cutoff membrane against 100 mM carbonate-bicarbonate buffer, pH 9.6, as described earlier [12,14]. This was followed by incubation with 100 *μ*L allergic or normal serum (1:3 dilution). Alkaline phosphatase-conjugated mouse antihuman IgE (Sigma-Aldrich, St. Louis, MO) was used as the secondary antibody (1:1000 dilution). The absorbance was measured at 405 nm using *p*-nitrophenyl phosphate as the substrate. When the A_405 _values were 2-fold higher than that of normal subject's sera (serum pooled from 5 nonatopic subjects), the ELISA was considered positive.

### SDS-PAGE and IgE-Immunoblotting

The peel, pulp, fruit and cooked eggplant extracts were examined for their protein patterns by SDS-PAGE (12%, reducing) and IgE-immunoblotting as per described methods [23,24]. Ten micrograms of protein sample from each extract was diluted in sample buffer containing 5% *β*-mercaptoethanol, denatured at 100°C for 10 minutes and run on SDS-PAGE gels. After electrophoresis, the gels were fixed for 45 minutes and silver stained. The size of the protein bands mentioned in SDS-PAGE and immunoblots hereafter refer to their relative molecular mass (M_r_).

Allergenic profiling was carried out using sera from 6 eggplant-allergic subjects showing positive SPT to peel, pulp, raw fruit and cooked extracts of eggplant. Approximately 40 *μ*g of protein from each of the different components was used for electrophoretic transfer onto nitrocellulose membranes after SDS-PAGE. After blocking, the membrane was incubated with allergic or normal serum [1:3 diluted in Trisbuffered saline/1% BSA/0.05% Tween-20, (TBS-T)] at 4°C overnight. The secondary antibody used was ALP-conjugated mouse antihuman IgE (Sigma-Aldrich) at 1:1000 dilution. The immunoblots were developed using the insoluble substrate BCIP-NBT (1: 5 dilutions in TBS-T without BSA).

### Detection of Glycoprotein by Periodic Acid-Schiff Staining and Lectinoblot Analysis

The eggplant peel extract was subjected to SDS-PAGE. After electrophoresis, one half was stained for glycoproteins by periodic acid-Schiff (PAS) staining [25] and the other half was transferred onto nitrocellulose membrane for glycoprotein detection using Con A-biotin conjugate and avidin-ALP reagents [26]. Ovalbumin (a glycoprotein) and bovine serum albumin or BSA (a nonglycoprotein) were used as positive and negative controls, respectively.

### Simulated Gastric Fluid Digestion Assay

Proteins from eggplant peel extract were digested in simulated gastric fluid (SGF) according to a previously published protocol [27]. Briefly, eggplant peel extract containing 280 *μ*g protein was dissolved in 60 *μ*L of prewarmed distilled water. Twenty microliters of prewarmed 0.4 M HCl containing 8 g/L of NaCl and 1.28% of pepsin (specific activity: 2650 U/mg protein; Sigma-Aldrich), were added. Digestion proceeded at 37°C with continuous shaking. Similarly, BSA, a protein that is considered to have a lesser allergenic potential,[28] was also subjected to SGF digestion as a control representing minor allergen. SGF-treated and untreated protein samples were incubated separately, and the extent of pepsinolysis followed with time (0, 1, 10, 30, 60, and 120 minutes). At each time point an aliquot (20 *μ*L) of the digest was periodically withdrawn, digestion was stopped with 6 *μ*L of 0.2 M Na_2_CO_3_, and samples were mixed with 6.5 *μ*L of 5 × sample buffer for SDS-PAGE analysis. The contents of the reaction were subsequently heated at 95°C for 5 minutes. Fifteen microliters of each sample were loaded per lane (~20 *μ*g protein per well) for separation by SDS-PAGE; one set was visualized by silver staining and the other set (~40 *μ*g protein per well) was transferred onto nitrocellulose membrane for IgE-immunoblot experiments with pooled eggplant-allergic sera.

## Results

### SPT and Allergen-Specific IgE Measurements

The wheal/flare diameter (in millimeters) obtained in SPT for the 6 study subjects were in the range of 4.5-6.0/16-30, 4.0-4.5/0-16, and 4.5-5.0/16-20 for eggplant peel, pulp, and raw eggplant (raw) extracts, respectively (Table 2). However, the wheal/flare response was definitely lower with cooked eggplant extract (3.0-4.0/0 mm) when compared with raw eggplant extract. Though there was considerable reduction in the wheal/flare diameters in the case of pulp extract compared with peel extract, the difference, however, was statistically nonsignificant. Further, slightly lower wheal/flare response was observed by prick-by-prick test compared with SPT for both eggplant peel and pulp in all the study subjects; the prick-by-prick response was in the range of 3.0-3.5 mm without any noticeable flare (Table 2). The wheal/flare diameters for peel and pulp by the prick-by-prick method were of similar magnitude.

**Table 2:** Results of SPT and Prick-By-Prick Test to Extracts From Different Parts of Eggplant Fruit

	Wheal/Flare Diameter (mm)						
	
	SPT					Prick-By-Prick Test	
		
Subject's ID	Fruit Extract (Raw)	Fruit Extract (Cooked)	Pulp Extract	Peel Extract	PC*	Peel	Pulp
A1	4.5/20	3.5/0	4/12	5/20	5/25	3.5/0	3.5/0
A2	4.5/20	3.0/0	4/0	4.5/16	6/20	3.5/0	3/0
A3	4.5/16	3.5/0	4.5/0	6/30	5/20	3.5/0	3.5/0
A4	4.5/20	4.0/0	4/10	5/30	6/30	3/0	3/0
A5	5/20	4.0/0	4.5/16	5/25	6/30	3.5/0	3.5/0
A6	4.5/20	3.5/0	4.5/12	5/25	5/25	3/0	3/0

*PC = positive control: histamine. 2HCl, 1.66 mg/mL (equivalent to 1 mg/mL histamine base); a wheal/flare reaction of 0-1/0 mm was seen in all the subjects for the negative control (50% glycerinated PBS).

Serum allergen-specific IgE for the peel, pulp, raw and cooked eggplant extracts was positive in all the 6 eggplant-allergic subjects, as revealed by significantly higher A_405 _values compared to the value for normal subjects (*P *< 0.05) (Table 3). Although the A_405 _values for eggplant peel were slightly higher than that of the pulp, the difference was statistically nonsignificant (*P *= 0.219). Similarly, there was no significant difference between raw and cooked eggplant extracts in terms of specific IgE (A_405_) values (Table 3).

**Table 3:** Results of Allergen-Specific IgE to Extracts From Different Parts of Eggplant Fruit

	ELISA Units (A_405_)*			
	
Subject's ID	Fruit (Raw)	Fruit (Cooked)	Pulp	Peel
A1	0.433	0.391	0.376	0.501
A2	0.616	0.522	0.414	0.579
A3	0.594	0.436	0.401	0.524
A4	0.473	0.397	0.399	0.539
A5	0.482	0.408	0.385	0.525
A6	0.516	0.442	0.433	0.612
NS†	0.196	0.188	0.184	0.212

*Mean of triplicate determinations. Amount of protein coated per well, 25 *μ*g; serum dilution, 1:3; secondary antibody dilution, 1: 1000.

†NS = pooled normal sera.

### Allergenic Localization in Eggplant Fruit

**Figure 1:**
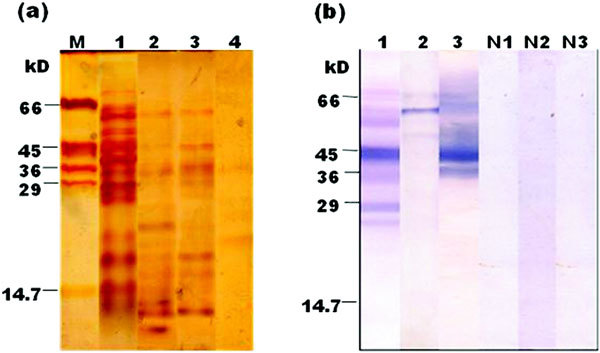
**(a) SDS-PAGE (12%, reducing) of extracts from different parts of eggplant fruit**. Protein load: 15 *μ*g; visualization: silver staining. M, Molecular weight markers; lane 1, eggplant peel extract; lane 2, eggplant pulp extract; lane 3, eggplant fruit (raw) extract; lane 4, cooked eggplant extract. (b) IgE-immunoblot using extracts from different parts of eggplant fruit and pooled sera from eggplant-allergic subjects. Lane 1, eggplant peel extract; lane 2, eggplant pulp extract; lane 3, eggplant fruit (raw) extract. N1, N2, and N3 denote eggplant peel, pulp, and fruit (raw) extracts, respectively, probed using pooled normal human sera.

The protein content of extracts from different parts of eggplant fruit are as follows: 26 mg/100 g eggplant peel, 19 mg/100 g eggplant pulp, 21 mg/100 g eggplant fruit (raw), and 2.4 mg/100 g eggplant fruit (cooked), all expressed on fresh weight basis (data not shown). The protein patterns of the eggplant peel, pulp, raw, and cooked eggplant extracts are represented in Figure 1 (panel a). SDS-PAGE (reducing) of eggplant peel extract showed approximately 30 silver-stained bands between 6 and 80 kD, whereas ~18 bands were observed between 3 and 71 kD in pulp extract. Twenty-two bands in the range of 5 to 80 kD were observed in raw eggplant extract, whereas only 2 protein bands (20 and 36 kD) were prominent in the cooked eggplant extract in addition to 2 faint bands at ~26 and 28 kD. Though the 20 kD band was absent in raw eggplant extract, it is present in cooked eggplant extract.

When immunoblots were performed using raw eggplant extract and individual eggplant-allergic sera, the IgE-binding patterns in all the 6 cases were similar to the pattern observed with raw eggplant extract using pooled eggplant-allergic sera shown in Figure 1 (panel b, lane 3). Because of lack of sufficient sera for testing the different eggplant extracts with individual serum, pooled sera from eggplant-allergic subjects were used. The IgE-immunoblots performed with eggplant peel, pulp, and raw eggplant extracts are shown in Figure 1 (panel b). Sera pooled from all the 6 eggplant-sensitized subjects detected 9 IgE-reactive bands (26, 28, 36, 43, 45, 54, 60, 64, and 71 kD; lane 1) in the peel extract whereas the pulp extract showed only 3 IgE-reactive bands (52, 60, and 71 kD; lane 2). However, raw eggplant extract showed 5 IgE-reactive bands of 36, 45, 60, 64, and 71 kD (lane 3). IgE-immunoblot performed with cooked eggplant extract did not reveal any protein bands (data not shown), though the SDS-PAGE profile (silver staining) of cooked eggplant extract (lane 4 of Figure 1, panel a) revealed that the 20, 26, 28, and 36 kD proteins appeared to be stable after cooking, of which the 26, 28, and 36 kD proteins are known to be IgE-reactive in the raw extracts; presence of some protein bands around 66 kD was also fairly evident in cooked eggplant extract.

**Figure 2:**
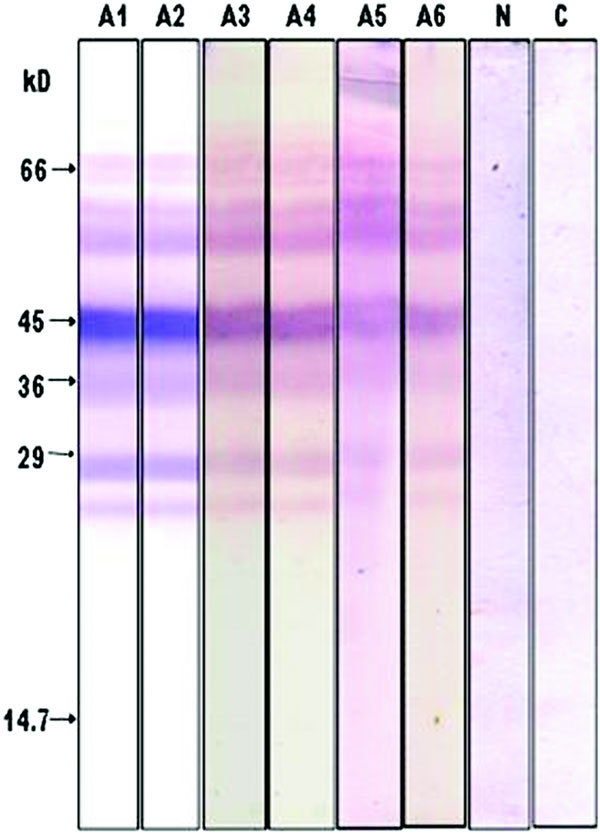
**IgE-immunoblot of eggplant peel extract using individual eggplant-allergic serum**. Lanes A1 to A6 represent immunoblots probed with the individual serum from eggplant-allergic subjects A1 to A6, respectively. N, immunoblot probed with pooled normal sera. C, Control immunoblot probed directly with the secondary antibody conjugate (murine monoclonal antihuman IgE-ALP) without the use of normal sera to demonstrate the lack of nonspecificity in the immunoblot patterns.

From the foregoing analysis, it is clear that eggplant peel contains the majority of the allergens found in eggplant fruit. Therefore, eggplant peel extract was subjected to immunoblotting and probed with individual serum of eggplant-allergic subjects. The results are shown in Figure 2. The immunoblotting pattern is identical in all the 6 cases; only in the case of serum from subject A5, there seems to be an additional high molecular weight band in the region around 90-100 kD (lane A5). One characteristic feature of the immunoblot with peel extracts is that the doublet around 43-45 kD is very intense. Incubation of eggplant peel extract directly with secondary antibody-ALP conjugate did not reveal any protein bands indicating the absence of nonspecific binding (lane C).

### Glycoprotein Profile of Eggplant Allergens

Because all the allergens present in eggplant pulp and eggplant fruit (raw) with the exception of 52 kD were also present in the eggplant peel, we analyzed the allergens present in eggplant peel extract for the presence of glycans in the allergenic proteins and, subsequently, their in vitro gastric stability using SGF.

**Figure 3:**
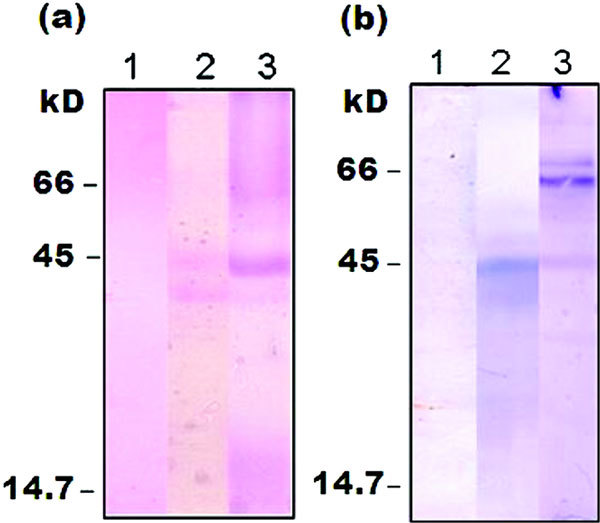
**(a) PAS staining of eggplant peel extract analyzed by SDS-PAGE (12%, reducing)**. Lane 1, BSA (a nonglycoprotein as negative control); lane 2, ovalbumin (a glycoprotein as positive control); lane 3, eggplant peel extract. Protein load: 15 *μ*g in all cases. (b) Detection of glycoproteins in eggplant peel extract by lectin-blot analysis using Con A-biotin conjugate followed by avidin-ALP conjugate on nitrocellulose. Lane 1, BSA (a nonglycoprotein as negative control); lane 2, ovalbumin (a glycoprotein as positive control); lane 3, eggplant peel extract. Protein load: 25 *μ*g in all cases.

PAS staining after SDS-PAGE of the eggplant peel extract detected the 43 and 45 kD as prominent glycoprotein bands whereas the 64 and 71 kD allergenic proteins appeared as faint, but sharp bands indicating the presence of glycans in all the 4 proteins (Figure 3, panel a). This was further confirmed by lectinoblot analysis of the eggplant peel extract by Con A-biotin/avidin-ALP detection system which detected 45, 64, and 71 kD allergens as glycoproteins recognized by Con A (Figure 3, panel b) indicative of the presence of mannose residues (mostly *N*-linked glycans) in these glycoproteins.

### Stability of Eggplant Allergens in SGF

**Figure 4:**
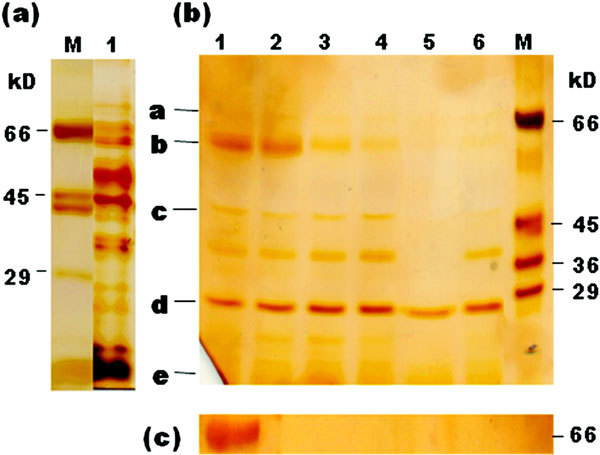
**Digestibility of proteins in eggplant peel extract in simulated gastric fluid as monitored by SDS-PAGE analysis (12%, reducing; silver staining)**. (a), Untreated eggplant peel extract shown in lane 1; (b), SGF-treated eggplant peel extract: lane 1, 0 minutes; lane 2, 1 minute; lane 3, 10 minutes; lane 4, 30 minutes; lane 5, 60 minutes; lane 6, 120 minutes. Protein bands labeled a-e represent the persistent parent proteins or protein fragments in SGF digestion as shown in Figure 5; (c), SGF-treated BSA, a protein that is considered to have lesser-allergenic potential; M, molecular weight markers.

SDS-PAGE of SGF-digested eggplant peel extract showed that the 26 kD (band d), 38 kD, 64 kD (band b), and 71 kD (band a) allergens appeared as faint but intact protein bands at 120 minutes of digestion, whereas the 45 kD allergen band (band c) remained intact up to 30 minutes and disappeared later as visualized by silver staining (Figure 4, panel b). In contrast, BSA (Bos d 6), generally considered as a minor allergen or a protein that is considered to have a lesser allergenic potential [28] was completely degraded within 1 minute of incubation (Figure 4, panel c).

**Figure 5:**
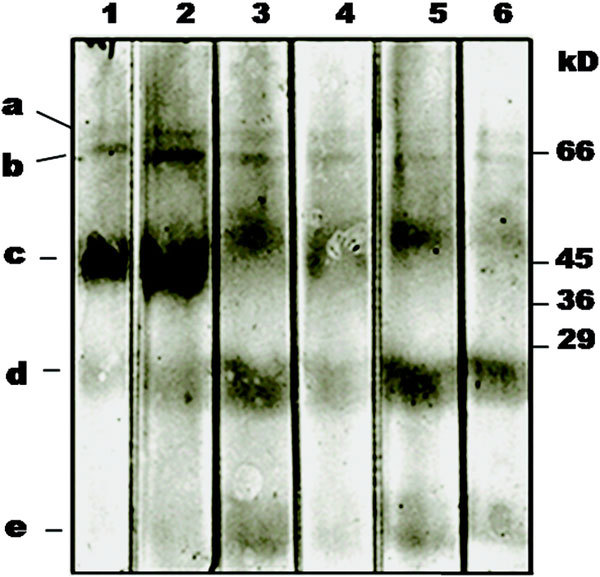
**IgE-immunoblot analysis of eggplant peel extract after SGF digestion using pooled sera from eggplant-allergic patients**. Kinetics of digestion: lane 1, 0 minutes; lane 2, 1 minute; lane 3, 10 minutes; lane 4, 30 minutes; lane 5, 60 minutes; lane 6, 120 minutes. Protein bands labeled a-e represent the persistent parent proteins or protein fragments in SGF digestion.

The IgE-immunoblots of SGF-digested eggplant peel extract probed with pooled eggplant allergic sera showed that the 64 and 71 kD allergens (labeled as band b and a, respectively) displayed uniform IgE-binding at all time intervals from 0 to 120 minutes of digestion (Figure 5). Protein band c, representing proteins around 45 kD, disappeared fairly rapidly after 1 minute of digestion, but persisted up to 120 minutes. Further, we also observed fairly good IgE-binding at 26 and 28 kD regions (labeled as band d indicating a doublet) in the IgE-immunoblots at 60 and 120 minutes of digestion. The 38 kD band though appeared stable at 120 minutes in SDS-PAGE of SGF-treated eggplant peel extract (Figure 4, panel b), was not detected in IgE-immunoblots. Protein band e represents several low molecular weight fragments of parent allergens appearing after 10 minutes of digestion; they seemed to retain IgE-binding.

## Discussion

Eggplant is known to induce IgE-mediated hypersensitive reactions in sensitized individuals with a wide spectrum of symptoms varying from skin rashes, wheezing, itching of the eye, gastrointestinal abnormalities, and itching and hoarseness of the throat; prevalence is ~0.8% having a female predominance [12,14,15]. Only 2 cases of anaphylaxis to eggplant have been described so far, one of them in a patient with latex allergy [13,16]. In this study, an attempt has been made to study the localization of allergens in different parts of eggplant fruit (peel and pulp) using sera from 6 eggplant-allergic subjects. The effect of cooking on the allergenicity of eggplant, and the presence of glycans in the allergenic proteins and their gastric stability in vitro were assessed.

The most common clinical presentation of eggplant allergy in the study subjects is either localized or generalized skin rashes (urticaria) affecting different parts of the body within 1-2 hours of ingestion of eggplant in cooked form. In the present study, 4 of 6 eggplant-allergic subjects had other atopic conditions mostly related to respiratory allergies. Generally, food allergy tends to occur in subjects who also have other inhalant allergies, but not always so [29]. Eggplant has recently been found to be a common food causing sensitization in respiratory allergic patients based on SPT diagnosis [30]. Two subjects (A3 and A5) who had sensitization to eggplant since early childhood did not outgrow eggplant allergy after reaching adolescence. Other food allergies were noted in 3 eggplant-allergic subjects among whom 2 subjects (A1 and A5) also have allergy to potato and tomato. The sensitization data clearly indicates that there could be possible cross-reactivity between eggplant and other foods of Solanaceae and non-Solanaceae families [7,13,16,31-33].

The SPTs with eggplant peel produced more pronounced wheal/flare response compared with pulp, indicating different parts of eggplant fruit can have different allergenicities. A similar trend was noticed in the case of SPTs with peel and pulp extracts of Rosaceae fruits, figs, and tomato [34-37]. Unlike in the case of tomato,[36] prick-by-prick method of skin testing was less effective in the case of eggplant probably because of a less watery nature and somewhat leathery surface of the eggplant fruit. Prick-by-prick method seems to be the effective in the case of fruits and those vegetables with a liquid nature. Cooked eggplant extract produced a slightly lower SPT response than the raw eggplant extract. These results are in good agreement with earlier reports on 4 cases of eggplant allergy [12,13]. Although histamine is present in eggplant (0.89 to 2.41 mg/100 g fresh weight of different varieties), its content in 1:10 wt/vol allergenic extracts used for SPT does not contribute to the wheal/flare response in a majority of nonatopic subjects [38].

All the eggplant-sensitized subjects have shown specific IgE to both eggplant peel and pulp extracts, indicating the presence of IgE-binding components. The relatively higher specific-IgE values for eggplant peel than the pulp further demonstrate varying allergenicities of different parts of eggplant fruit. Allergen-specific IgE to both raw and cooked eggplant extracts showed similar IgE-binding patterns comparable to earlier studies,[12,13] which further substantiates the results of SPT.

Immunoblotting with pooled eggplant-allergic sera detected 9 IgE-binding proteins between 26-71 kD in eggplant peel, and 3 major IgE-binding proteins between 52-71 kD in eggplant pulp. However, in the raw eggplant extract, 5 IgE-binding proteins between 36-71 kD were detected. This clearly indicates that most of the allergens are concentrated in the eggplant peel. The prevalence of allergenic proteins in the external layer such as peel is a common feature in the case of figs (specifically, the syconium of figs) and other allergenic fruits of the Rosaceae family such as apple, peach, and pear [34,35,39,40]. It has been shown recently that the content of the lipid transfer protein, Pru p 3 (a major peach fruit allergen) is approximately 250-fold higher in the peel as compared with the pulp on a per g fresh weight basis [39]. Immuno-tissue-print analyses have revealed that Mal d 1 and Mal d 2 are distributed throughout the apple pulp and peel, whereas Mal d 3 (a 9 kD nonspecific lipid transfer protein) is restricted to the peel [40]. It should be recalled here that only 3 allergens (60, 64, and 71 kD) were mainly detected in the first description of eggplant allergy wherein only raw eggplant extract (representing peel to pulp ratio of approximately 1:9) was used [12].

Gel electrophoresis by silver staining revealed the presence of 4-6 protein bands in cooked eggplant extract; it may be noted here that the 71 kD protein band was identified as a heat-stable protein in our earlier study [12]. However, immunoblotting with cooked eggplant extract was not successful because of technical problems such as low amounts of protein (one-tenth of that of raw eggplant extract) and viscous nature because of the high amount of carbohydrate polymers in the pulp [1]. In the study by Lee et al, out of the 3 allergens detected between 22 and 50 kD, the protein band between 22 and 36 kD showed cross-reactivity with latex [13].

Among the allergenic components identified in the eggplant peel, the 45, 64, and 71 kD allergens are glycoproteins as seen in lectinoblots using Con A, a lectin having mannose/glucose specificity. Although the 43 kD protein showed glycoprotein nature by PAS-staining, its nondetection in lectinoblots could possibly be because of the absence of mannose residues in the glycoprotein. Because many of the cross-reactive food allergens are glycated in nature,[9,33] it may be presumed that the reported cross-reactivity of eggplant with pollens, latex, other Solanaceae and non-Solanaceae foods may be because of the similarity of eggplant glycoproteins to other reported glycoprotein allergens [6,13,31-33,41-43]. Several allergens like profilin, pathogenesis-related protein P23 and Bet v 1 have been described in bell pepper [44]. However, the cross-reactivity studies of eggplant-allergic sera with other vegetables of the Solanaceae family (tomato, potato, and bell pepper) requires a detailed investigation by immunoblot inhibition.

The relatively uniform IgE-binding of the 3 allergens (26, 64, and 71 kD) in eggplant peel after SGF digestion further strengthens the point that the allergenic proteins exhibit persistence in gastric environment that has been considered as an important criterion for allergenicity assessment [45,46]. In fact, it is suggested that in vitro digestion protocols should be combined with immunologic assays to elucidate the role of large digestion-resistant fragments and the influence of the food matrix on the stimulation of the immune system [45]. The reason for nonbinding of serum IgE to the 36 kD band allergen after SGF treatment is not clear. Further, it may be noted that the proteins present in the fruit and vegetables generally exhibit gastric stability compared with purified proteins, possibly because of the matrix-effect in fruits and vegetables that are rich in pectins as observed in the case of kiwi fruit [47].

Several plant allergens correspond to proteins having a defense role in plants [48]. Because the peel acts as a barrier for the invasion by predators, pathogens or parasites present in the external environment, its content of defense proteins should be quantitatively or qualitatively different from that of the internal pulp [34]. According to the results obtained here, this view seems to apply to eggplant also in view of the significant number of allergens in the peel though the peel amounts to approximately 10% of eggplant fruit on a fresh weight basis. However, most culinary preparations involve using the whole eggplant fruit without the removal of the thin edible peel. It should be noted here that allergic reactions appeared more frequently and were more severe when the whole fruit (in case of Rosaceae) was eaten [34]. More than 40% of patients allergic to apple and pear tolerate the ingestion of the pulp of these fruits, and reactions were only elicited by the intake of the whole fruit; peels induced higher SPTs, histamine release, and radio-allergosorbent tests than pulps [34]. A careful recording of case history should also include attention to whether the eggplant was peeled or not in the culinary preparations. The identification of eggplant allergens remains a challenging task in view of the low protein content (1% of fresh weight) and multitude of proteins; purification to homogeneity of detected allergens by classic protein purification methods for further characterization appears to pose significant technical problems. In conclusion, the results of the present study clearly demonstrate that eggplant is a multiallergenic vegetable in the context of presence of allergens in all edible parts of eggplant with a predominant localization in the peel.

## End Note

Bheemanapalli N. Harish Babu, MSc (Agri), is currently at All India Coordinated Research Project (Safflower), Agricultural Research Station, Annigeri-582201, Dharwad District, Karnataka State, India.

Source of support: Grant-in-aid project (No. BT/PR6281/AGR/16/574/2005) to Y.P.V. from the Department of Biotechnology, Government of India, New Delhi, India. Senior research fellowship to B.N.H. from the Council of Scientific & Industrial Research, New Delhi, India.

## References

[B1] Lawande KF Chavan JK Salunkhe DK, Kadam SS Eggplant (Brinjal) Handbook of Vegetable Science and Technology: Production, Composition, Storage, and Processing 1998 New York: Marcel Dekker 225 244

[B2] Kashyap V Kumar SV Collonnier C Fusari F Haicour R Rotino GL Sihachakr D Rajam MV Biotechnology of eggplant Sci Hortic 2003 2 1 25 10.1016/S0304-4238(02)00140-1

[B3] Lans C Comparison of plants used for skin and stomach problems in Trinidad and Tobago with Asian ethnomedicine J Ethnobiol Ethnomed 2007 2 3 [doi: 10.1186/1746-4269-3-3] 10.1186/1746-4269-3-3 17207273 PMC1781930

[B4] Seppälä U Alenius H Turjanmaa K Reunala T Palosuo T Kalkkinen N Identification of patatin as a novel allergen for children with positive skin prick test responses to raw potato J Allergy Clin Immunol 1999 2 165 171 10.1016/S0091-6749(99)70541-5 9893201

[B5] Seppälä U Majamaa H Turjanmaa K Helin J Reunala T Kalkkinen N Palosuo T Identification of four novel potato (*Solanum tuberosum*) allergens belonging to the family of soybean trypsin inhibitors Allergy 2001 2 619 626 10.1034/j.1398-9995.2001.00058.x 11421919

[B6] Schmidt MH Raulf-Heimsoth M Posch A Evaluation of patatin as a major cross-reactive allergen in latex-induced potato allergy Ann Allergy Asthma Immunol 2002 2 613 618 10.1016/S1081-1206(10)62110-2 12487228

[B7] Foetisch K Westphal S Lauer I Retzek M Altmann F Kolarich D Biological activity of IgE specific for cross-reactive carbohydrate determinants J Allergy Clin Immunol 2003 2 889 896 10.1067/mai.2003.173 12704374

[B8] Kondo Y Urisu A Tokuda R Identification and characterization of the allergens in the tomato fruit by immunoblotting Int Arch Allergy Immunol 2001 2 294 299 10.1159/000049526 11815736

[B9] Westphal S Kolarich D Foetisch K Lauer I Altmann F Molecular characterization and allergenic activity of Lyc e 2 (beta-fructo-furanosidase), a glycosylated allergen of tomato Eur J Biochem 2003 2 1327 1337 10.1046/j.1432-1033.2003.03503.x 12631291

[B10] Willerroider M Fuchs H Ballmer-Weber BK Focke M Susani M Cloning and molecular and immunological characterization of two new food allergens, Cap a 2 and Lyc e 1, profilins from bell pepper (*Capsicum annuum*) and tomato (*Lycopersicum esculentum*) Int Arch Allergy Immunol 2003 2 245 255 10.1159/000072136 12915767

[B11] Wagner S Radauer C Hafner C Fuchs H Jensen-Jarolim E Characterization of cross-reactive bell pepper allergens involved in the latex-fruit syndrome Clin Exp Allergy 2004 2 1739 1746 10.1111/j.1365-2222.2004.02103.x 15544599

[B12] Pramod SN Venkatesh YP Allergy to eggplant (*Solanum melongena*) J Allergy Clin Immunol 2004 2 171 173 10.1016/j.jaci.2003.10.037 14713925

[B13] Lee J Cho YS Park SY Lee CK Yoo B Moon HB Park HS Eggplant anaphylaxis in a patient with latex allergy J Allergy Clin Immunol 2004 2 995 996 10.1016/j.jaci.2004.01.565 15148961

[B14] Pramod SN Venkatesh YP Allergy to eggplant (*Solanum melongena*) caused by a putative secondary metabolite J Investig Allergol Clin Immunol 2008 2 59 62 18361104

[B15] Harish Babu BN Mahesh PA Venkatesh YP A cross-sectional study on the prevalence of food allergy to eggplant (*Solanum melongena *L.) reveals female predominance Clin Exp Allergy 2008 2 1795 1802 18681854 10.1111/j.1365-2222.2008.03076.x

[B16] Muthiah R Zondlo A Zondlo J Kagen S Eggplant anaphylaxis: isolation of the major allergens and demonstration of cross-reactivity with other Solanaceae J Allergy Clin Immunol 1996 2 334 [Abstract]

[B17] Larramendi CH Ferrer A Huertas AJ García-Abujeta JL Andreu C Sensitization to tomato peel and pulp extracts in the Mediterranean coast of Spain. Prevalence and co-sensitization with aeroallergens Clin Exp Allergy 2008 2 169 177 18005185 10.1111/j.1365-2222.2007.02865.x

[B18] Bradford MM A rapid and sensitive method for the quantitation of microgram quantities of protein utilizing the principle of protein-dye binding Anal Biochem 1976 2 248 254 10.1016/0003-2697(76)90527-3 942051

[B19] Pongsakul N Leelasart B Rakariyatham N Effect of L-cysteine, potassium metabisulfite, ascorbic acid and citric acid on inhibition of enzymatic browning in longan Chiang Mai J Sci 2006 2 137 141

[B20] Sanico AM Bochner BS Saini SS Adelman DC, Casale TB, Corren J Skin testing methods Manual of Allergy and Immunology 2002 4 Philadelphia, PA: Lippincott Williams & Wilkins 485 486

[B21] Cantani A Micera M The prick by prick test is safe and reliable in 58 children with atopic dermatitis and food allergy Eur Rev Med Pharmacol Sci 2006 2 115 120 16875044

[B22] Hamilton RG Adkinson NF Jr Clinical laboratory assessment of IgE-dependent hypersensitivity J Allergy Clin Immunol 2003 2 S687 S701 10.1067/mai.2003.123 12592314

[B23] Laemmli UK Cleavage of structural proteins during the assembly of the head of bacteriophage T_4_ Nature 1970 2 680 685 10.1038/227680a0 5432063

[B24] Towbin HK Staehelin TH Gordon J Electrophoretic transfer of proteins from polyacrylamide gels to nitrocellulose sheets Proc Natl Acad Sci USA 1979 2 4350 4354 10.1073/pnas.76.9.4350 388439 PMC411572

[B25] Zacharius RM Zell TE Morrison JH Woodlock JJ Glycoprotein staining following electrophoresis on acrylamide gels Anal Biochem 1969 2 148 152 10.1016/0003-2697(69)90383-2 4183001

[B26] Duk M Lisowska E Wu JH Wu AM The biotin/avidin-mediated microtiter plate lectin assay with the use of chemically modified glycoprotein ligand Anal Biochem 1994 2 266 272 10.1006/abio.1994.1410 7810865

[B27] Yagami T Haishima Y Nakamura A Osuna H Ikezawa Z Digestibility of allergens extracted from natural rubber latex and vegetable foods J Allergy Clin Immunol 2000 2 752 756 10.1067/mai.2000.109171 11031347

[B28] Herman RA Woolhiser MM Ladics GS Korjagin VA Schafer BW Stability of a set of allergens and non-allergens in simulated gastric fluid Int J Food Sci Nutr 2007 2 125 141 10.1080/09637480601149640 17469768

[B29] Schäfer T Böhler E Ruhdorfer S Weigl L Wessner D Epidemiology of food allergy/food intolerance in adults: associations with other manifestations of atopy Allergy 2001 2 1172 1179 10.1034/j.1398-9995.2001.00196.x 11736746

[B30] Mandal J Das M Roy I Chatterjee S Barui NC Gupta-Bhattacharya S Immediate hypersensitivity to common food allergens: an investigation on food sensitization in respiratory allergic patients of Calcutta, India World Allergy Org J 2009 2 9 12 10.1097/WOX.0b013e318194c0de PMC3650995 23282888

[B31] Reche M Pascual CY Vicente J Caballero T Martin-Munoz F Sanchez S Martin-Esteban M Tomato allergy in children and young adults: cross-reactivity with latex and potato Allergy 2001 2 1197 1201 10.1034/j.1398-9995.2001.00279.x 11736750

[B32] Gamboa PM Sanchez-Monge R Diaz-Perales A Salcedo G Ansotegui J Sanz ML Latex-vegetable syndrome due to custard apple and aubergine: new variations of the hevein symphony J Investig Allergol Clin Immunol 2005 2 308 311 16433216

[B33] Palomares O Villalba M Quiralte J Polo F Rodriguez R 1,3-betaglucanases as candidates in latex-pollen-vegetable food cross-reactivity Clin Exp Allergy 2005 2 345 351 10.1111/j.1365-2222.2004.02186.x 15784114

[B34] Fernández-Rivas M Cuevas M Peels of Rosaceae fruits have a higher allergenicity than pulps Clin Exp Allergy 1999 2 1239 1247 10.1046/j.1365-2222.1999.00628.x 10469033

[B35] Antico A Zoccatelli G Marcotulli C Curioni A Oral allergy syndrome to fig Int Arch Allergy Immunol 2003 2 138 142 10.1159/000070929 12811022

[B36] Ferrer A Huertas AJ Larramendi CH García-Abujeta JL Bartra J Usefulness of manufactured tomato extracts in the diagnosis of tomato sensitization: comparison with the prick-prick method Clin Mol Allergy 2008 2 1 [doi: 10.1186/1476-7961-6-1] 10.1186/1476-7961-6-1 18184431 PMC2263074

[B37] Vandenplas O Sohy C D'Alpaos V Nootens C Thimpont J Tomato-induced occupational asthma in a greenhouse worker J Allergy Clin Immunol 2008 2 1229 1231 10.1016/j.jaci.2008.07.035 18790524

[B38] Kiran Kumar MN Harish Babu BN Venkatesh YP Higher histamine sensitivity in non-atopic subjects by skin prick test may result in misdiagnosis of eggplant allergy Immunol Invest 2009 2 93 103 10.1080/08820130802608295 19172488

[B39] Ahrazem O Jimeno L López-Torrejón G Herrero M Espada JL Assessing allergen levels in peach and nectarine cultivars Ann Allergy Asthma Immunol 2007 2 42 47 10.1016/S1081-1206(10)60619-9 17650828

[B40] Marzban G Puehringer H Dey R Brynda S Ma Y Martinelli A Localisation and distribution of the major allergens in apple fruits Plant Sci 2005 2 387 394 10.1016/j.plantsci.2005.03.027

[B41] Hofmann A Burks AW Pollen food syndrome: update on the allergens Curr Allergy Asthma Rep 2008 2 413 417 10.1007/s11882-008-0080-0 18682109

[B42] Ortega N Quiralte J Blanco C Castillo R Alvarez MJ Carrillo T Tobacco allergy: demonstration of cross-reactivity with other members of Solanaceae family and mugwort pollen Ann Allergy Asthma Immunol 1999 2 194 197 10.1016/S1081-1206(10)62596-3 10071524

[B43] Gubesch M Theler B Dutta M Baumer B Mathis A Holzhauser T Vieths S Ballmer-Weber BK Strategy for allergenicity assessment of 'natural novel foods': clinical and molecular investigation of exotic vegetables (water spinach, hyacinth bean and Ethiopian eggplant) Allergy 2007 2 1243 1250 10.1111/j.1398-9995.2007.01474.x 17919138

[B44] Jensen-Jarolim E Santner B Leitner A Grimm R Scheiner O Ebner C Breiteneder H Bell peppers (*Capsicum annuum*) express allergens (profilin, pathogenesis-related protein P23 and Bet v 1) depending on the horticultural strain Int Arch Allergy Immunol 1998 2 103 109 10.1159/000023932 9652302

[B45] Moreno FJ Gastrointestinal digestion of food allergens: effect on their allergenicity Biomed Pharmacother 2007 2 50 60 10.1016/j.biopha.2006.10.005 17188456

[B46] Schnell S Herman RA Should digestion assays be used to estimate persistence of potential allergens in tests for safety of novel food proteins? Clin Mol Allergy 2009 2 1 [doi: 10.1186/1476-7961-7-1] 10.1186/1476-7961-7-1 19146693 PMC2632610

[B47] Polovic N Blanusa M Gavrovic-Jankulovic M Atanaskovic-Markovicw M Burazerz L A matrix effect in pectin-rich fruits hampers digestion of allergen by pepsin in vivo and in vitro Clin Exp Allergy 2007 2 764 771 10.1111/j.1365-2222.2007.02703.x 17456224

[B48] Ebner C Hoffmann-Sommergruber K Breiteneder H Plant food allergens homologous to pathogenesis-related proteins Allergy 2001 2 Suppl 67 43 44 11298007 10.1034/j.1398-9995.2001.00913.x

